# Genetic Contribution to End-Stage Cardiomyopathy Requiring Heart Transplantation

**DOI:** 10.1161/CIRCGEN.123.004062

**Published:** 2023-09-28

**Authors:** Yuri Kim, Oddný Brattberg Gunnarsdóttir, Anissa Viveiros, Daniel Reichart, Daniel Quiat, Jon A. L. Willcox, Hao Zhang, Huachen Chen, Justin J. Curran, Daniel H. Kim, Simon Urschel, Barbara McDonough, Joshua Gorham, Steven R. DePalma, Jonathan G. Seidman, Christine E. Seidman, Gavin Y. Oudit

**Affiliations:** 1 Division of Cardiovascular Medicine, Brigham and Women's Hospital; 2 Department of Genetics, Harvard Medical School, Boston, MA; 3 Demartment of Medicine, University of Alberta; 4 Mazankowski Alberta Heart Institute, Edmonton, Alberta, Canada; 5 Department of Medicine I, University Hospital, Ludwig Maximilian University of Munich, Munich, Germany; 6 Department of Cardiology, Boston Children's Hospital, Boston, MA; 7 Department of Pediatrics, University of Alberta; 8 Stollery Children's Hospital, Edmonton, Alberta, Canada; 9 Howard Hughes Medical Institute, Chevy Chase, MD

## Abstract

**Background –:**

Many cardiovascular disorders propel the development of advanced heart failure that necessitate cardiac transplantation. When treatable causes are excluded, studies to define etiologies are often abandoned, resulting in a diagnosis of end-stage idiopathic cardiomyopathy. We studied whether DNA sequence analyses could identify unrecognized causes of end-stage non-ischemic cardiomyopathy requiring heart transplantation and whether the prevalence of genetic etiologies differed from ambulatory cardiomyopathy cases.

**Methods –:**

We performed whole exome and genome sequencing of 122 explanted hearts from 101 adult and 21 pediatric patients with idiopathic cardiomyopathy from a single center. Data were analyzed for pathogenic/likely pathogenic variants in nuclear and mitochondrial genomes and assessed for nonhuman microbial sequences. The frequency of damaging genetic variants was compared among cardiomyopathy cohorts with different clinical severity.

**Results –:**

Fifty-four samples (44.3%) had pathogenic/likely pathogenic cardiomyopathy gene variants. The frequency of pathogenic variants was similar in pediatric (42.9%) and adult (43.6%) samples, but the distribution of mutated genes differed (p=8.30e-04). The prevalence of causal genetic variants was significantly higher in end-stage than in previously reported ambulatory adult dilated cardiomyopathy cases (p<0.001). Among remaining samples with unexplained etiologies, no damaging mitochondrial variants were identified, but 28 samples contained parvovirus genome sequences, including 2 samples with 6 to 9-fold higher levels than the overall mean levels in other samples.

**Conclusions –:**

Pathogenic variants and viral myocarditis were identified in 45.9% of patients with unexplained end-stage cardiomyopathy. Damaging gene variants are significantly more frequent among transplant compared to ambulatory cardiomyopathy patients. Genetic analyses can help define cause of end-stage cardiomyopathy to guide management and risk stratification of patients and family members.

## Introduction

Heart failure (HF) is a devastating disorder with high morbidity and mortality, affecting at least 26 million people worldwide^1^ and leading to death in 50% of patients within 5 years after the first HF hospitalization^2^. Cardiomyopathy, a pathologic remodeling of the myocardium that precedes HF, is typically classified as ischemic or non-ischemic origin.

Genetic causes of non-ischemic cardiomyopathy are increasingly recognized^3,4^. Dilated (DCM), hypertrophic (HCM), and arrhythmogenic (ACM) cardiomyopathies are often monogenic disorders caused by damaging variants within protein coding sequences, or arise from polygenic risk variants^3^ and environmental factors^5^. A causal genetic variant is found in approximately 10–30% of sporadic DCM and 30–50% of HCM cases^6,7^. Genetic causes of DCM are more varied and scores of putative DCM genes have been hypothesized. The putative DCM genes are involved in various cellular functions within cardiomyocytes including contractile apparatus (sarcomeres), calcium cycling, nuclear membranes, heat shock chaperones, mitochondria, and cell adhesion^8^. Pathogenic variants in gene titin (*TTN*) is the most common genetic cause of adult-onset DCM, found in 15–20% of affected individuals, but rarely identified in childhood-onset DCM^9,10^. TTN serves as a scaffold to support formation of the contractile apparatus and a molecular spring that influences contractility and relaxation. Genetic variants in *TTN* that cause DCM by creating premature truncation of the protein are denoted TTNtv^11^. The second most common genetic cause of DCM are rare pathogenic variants in lamin A/C (*LMNA*), which encodes a ubiquitously expressed nuclear membrane protein that participates in the maintenance of nuclear structure. *LMNA* variants account for approximately 5% of DCM cases and have a higher prevalence among DCM patients with cardiac conduction abnormalities^12^.

Pathogenic variants that cause HCM generally occur in eight sarcomere genes encoding: myosin binding protein C (*MYBPC3*), myosin heavy chain (*MYH7*), regulatory myosin light chain (*MYL2*), essential myosin light chain (*MYL3*), troponin I (*TNNI3*), troponin T, (*TNNT2*), tropomyosin (*TPM1*), and alpha cardiac actin (*ACTC1*)^7,13^. Pathogenic variants in *MYBPC3* and *MYH7* predominate, accounting for up to 50% of all HCM cases and 75% of cases with a defined genetic cause^13^. About 3–8% of HCM patients develop end-stage HCM characterized by left ventricular ejection fraction (LVEF) < 50%. Approximately 30–50% of end-stage HCM patients develop refractory HF and 11–25% require heart transplant^14^.

ACM is characterized by myocardial atrophy and fibrofatty replacement of the myocardium that may predominately alter the right ventricle or cause biventricular dysfunction^15^. The prevalence of ACM is estimated to be 1:2000–1:5000 and typically follows an autosomal dominant inheritance pattern^16^ albeit with variable penetrance. Pathogenic variants in desmosome genes are the most common genetic variants in ACM and include plakophilin 2 (*PKP2*), desmoplakin (*DSP*), desmocollin 2 (*DSC2*), junction plakoglobin (*JUP*), and desmoglein 2 (*DSG2*)^17^.

Cardiomyopathies that arise during childhood and cause HF are rare and often lethal conditions^18^. The causes of cardiomyopathy in children are more diverse than in adult and include metabolic, neuromuscular, and syndromic genes^19^. Additionally, pathogenic variants in the mitochondrial genome, which occur in 1 in 5,000 newborns cause cardiomyopathies in 40% of cases, with high rates of early mortality^20^. As children with mitochondrial cardiomyopathies have greater rates of morbidities after transplantation, careful selection of appropriate candidates has been urged^21^. Acute myocarditis is a rare cause of childhood HF, but longitudinal studies indicate persistent myocardial dysfunction in almost 50% of cases, with almost 19% requiring cardiac transplantation^22^.

Despite the recognition of heritable^23^ and microbial^24^ causes of cardiomyopathies, little is known about the contribution of these to end-stage HF requiring transplant. A previous study from Spain reported causal genetic variants in 73% of 52 transplant patients with familial DCM patients, including 25% with a founder mutation in the emerin (*EMD*) gene^25^. Another study employed gene-panel testing of peripheral blood from 31 heart transplant patients with non-ischemic cardiomyopathy and identified pathogenic and likely pathogenic variants in 38.7%^26^.

Comprehensive cardiac tissue DNA analyses to interrogate germline nuclear, mitochondrial, and microbial sequences provide the opportunity to define causes of heretofore idiopathic HF. Therefore, we analyzed whole exome (WES) and genome (WGS) sequences of explanted non-ischemic cardiomyopathy tissues from heart transplant, regardless of family history of cardiomyopathy, to identify and define the prevalence of pathogenic variants in end-stage cardiomyopathy.

## Methods

This study was performed using de-identified codes and protocols that were reviewed and approved by the human research committees at University of Alberta (Edmonton, Canada) and Brigham and Women's Hospital at Harvard Medical School (Boston, USA). All patients and/or their family provided written informed consent. The data and analytic methods will be made available to other researchers upon request. Further details are provided in Supplemental Methods.

## Results

### Clinical characteristics of study population

We performed WES/WGS analyses on 122 explant tissues from patients with idiopathic cardiomyopathies undergoing orthotopic heart transplantation (Table 1). No patient had prior genetic analyses. Clinical diagnoses in 101 adult patients included DCM (n=85), HCM (n=13), and ACM (n=3). Twenty-one pediatric patients were diagnosed with DCM (n=14), HCM (n=5), and RCM (n=2). Adult patients were predominantly male (78.2%), self-identified as white (91.1%), and had mean ages at transplant of 50 years (DCM), 45 years (HCM), and 37 (ACM). Two DCM patients were related. Pediatric cases were 47.6% females, self-reported as non-white (42.9%), and had mean ages at transplant of 7.4 years (DCM), 2.2 years (HCM), and 4.2 years (RCM).

### Rare pathogenic variants in DCM explanted hearts

To determine contribution of genetic variants to end-stage DCM, we performed WES/WGS analysis of 99 explant DCM tissues from heart transplantation. From initial analyses of coding variants, we identified 42 pathogenic and likely pathogenic variants in 53 cardiomyopathy genes (Table I). These included 32 loss-of-function (LoF) variants, 7 missense variants, and 3 samples with large structural variants, each causing autosomal-dominant disease (Tables 2 and III–V). Damaging variants in TTN accounted 42.9% of genotype-positive DCM cases, followed by *LMNA* (16.7%), *BAG3* (7.1%), and *DSP* (7.1%) (Tables 2 and III–V). Two related individuals shared the same LoF variant in BAG cochaperone 3 (BAG3 p.Arg123*; Table III). We also identified 1 rare splicing and 6 rare damaging missense variants in posited DCM genes (Tables II and VII) with uncertain pathogenicity.

**Figure 1.**
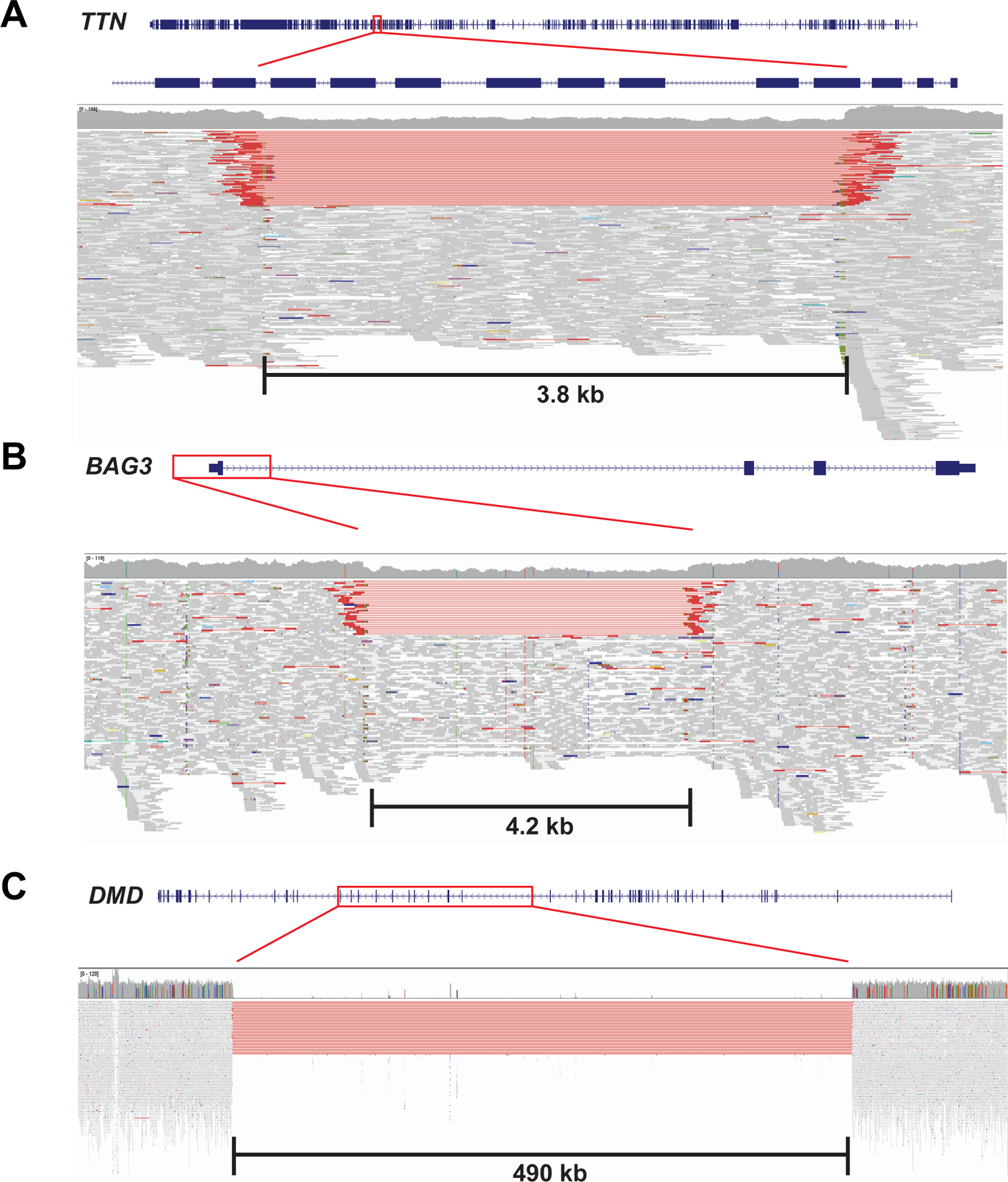
Large structural variants identified in DCM cases. **(A)** A 3.8 kb deletion in *TTN* involving seven exons leading to out-of-frame deletion. **(B)** A 4.2 kb deletion involving the promoter and exon 1 of *BAG3*, leading to haploinsufficiency. **(C)** A 490 kb deletion leading to hemizygous in-frame deletion of *DMD* in a male patient. Figures are modified from UCSC Genome Browser^28^ and Integrative Genomics Viewer^29^. Red box in each panel indicates location of the structural variant relative to each affected gene. Height of vertical thick grey bars in the middle of each panel indicates the number of sequence reads mapping to the reference genome at a particular location. Thin horizontal grey lines at the bottom of each panel represent sequencing reads aligned to sequences of each gene. Thin horizontal red lines demonstrate sequencing reads that span over a long distance within the genome, indicating the presence of large sequence deletions.

Several genes had atypical types of pathogenic variants. A structural variant in *TTN* (sample A0036) removes seven exons with high proportion spliced in (PSI)^27^ values and created an out-of-frame deletion (Figure 1A). A structural variant in *BAG3* (sample A0097) leads to deletion of the promoter and exon 1 of *BAG3* resulting in haploinsufficiency (Figure 1B), which was confirmed via RNA-seq analysis. Sample A0187's genome contains a large structural variant in dystrophin (*DMD*), which is predicted to encode a large in-frame deletion of DMD (Figure 1C). RNA-seq analyses of A0187 RNA confirmed the multi-exon loss in DMD RNA. Overall, next generation sequencing analysis enabled genetic diagnosis in 42 of 99 end-stage DCM cases (42.4%).

Genetic data altered three patients' clinical diagnosis of DCM. Patient A0127 carried a prototypical pathogenic HCM mutation (*MYBPC3* c.26–2A>G). The male patient (A0187) with an in-frame *DMD* had a history of chronically elevated creatinine kinase with negative evaluations for autoimmune myopathy/myositis and was reclassified with Becker muscular dystrophy. Female patient A0035 carried a heterozygous splice-site variant in lysosome-associated membrane protein 2 (*LAMP2*) that encoded a deletion and frameshift. Although males with LoF variants in X-linked *LAMP2* have Danon disease with massive cardiac hypertrophy, prevalent arrhythmias, and skeletal muscle, hepatic, and neurocognitive dysfunction, she like other women with damaging *LAMP2* variants^30^ had no extra-cardiac manifestations.

### Rare pathogenic variants in HCM, ACM, and RCM explanted hearts

Genetic analyses of 18 tissues from patients with clinical diagnosis of HCM identified causal variants in 50% of samples, including 3 LoF and 6 damaging missense variants (Table VIII). Among 13 adult patients 8 pathogenic variants were identified (61.5%) in sarcomere genes *MYBPC3* (5 variants), *MYH7* (2 variants), and *TNNT2* (1 variant). In contrast, only one of five pediatric HCM samples (20.0%) had a damaging variant, in a non-sarcomere gene *RIT1* (encoding Ras-like without CAAX protein-1). Activating *RIT1* variants cause Noonan syndrome, a finding that supports distinct genetic etiologies and molecular pathophysiology in pediatric- and adult-onset HCM^31^.

Among the few patients with ACM (n=3) and RCM (n=2), three causal genetic variants were identified. ACM variants included a LoF variant in *PKP2* and a missense founder variant TMEM43 p.Ser358Leu, predicted to have arisen in a Europe 1300–1500 years ago and recurrently observed in Canadian (Newfoundland) subjects^32^ (Tables 4 and IX). Both RCM cases were from pediatric patients, and a damaging missense variant in *MYH7* was found in one sample (Tables 5 and X).

### Analyses of mitochondrial variants and microbial sequences in explanted hearts

For 86 explanted tissues from 65 adult and 21 pediatric patients with unsolved cardiomyopathies after exome analyses, we examined mitochondrial sequences within WGS data. No pathogenic variants were identified with a heteroplasmic fraction^33^ > 0.1. Additionally, among patients of European ancestry, there was no mitochondrial haplotype enrichment compared to ancestry-matched controls.

**Figure 2.**
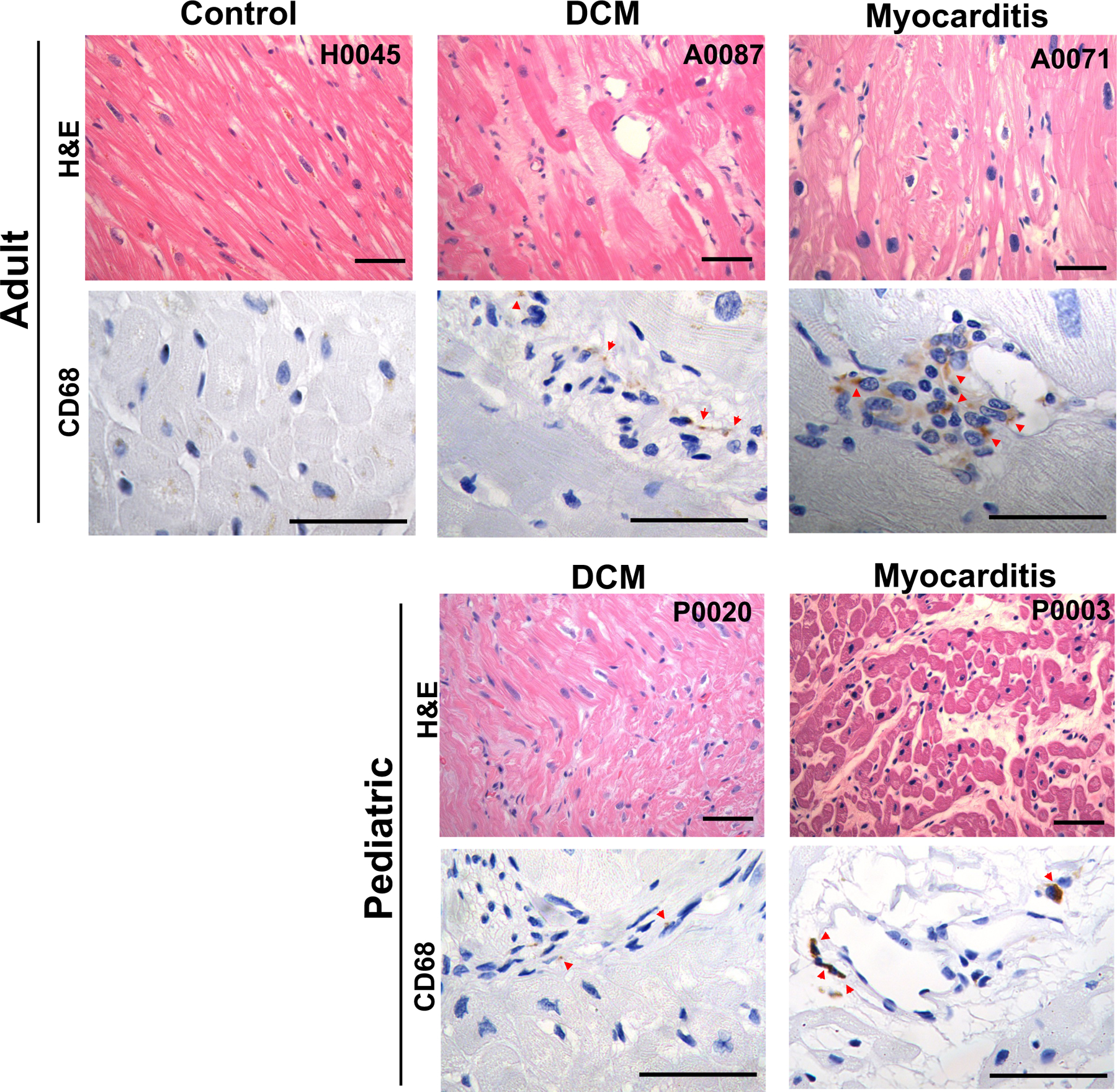
Myocardial histological staining of adult and pediatric cases with parvovirus infection. Histology of the human heart using hematoxylin and eosin (H&E) staining (top panels), and immunohistochemical (IHC) staining for CD68-positive macrophages (bottom panels). Staining was performed for control, dilated cardiomyopathy (DCM) and potential myocarditis cases from adult and pediatric donors (scale bar: 50 µm). Red arrows point to positive staining in IHC images.

We then examined WGS for the presence of genomic sequences of parvovirus B19, the most common cause of viral myocarditis^24^. Twenty-eight of 86 tissues carried sequences for parvovirus B19. Across all samples, the read counts encoding parvovirus B19 ranged from 2 to 197 (mean 21.9, median 9). Explanted tissues from two patients had strikingly higher viral read counts: 125 (A0071) and 197 (P0003). Patient A0071, an adult with DCM, had cardiac magnetic resonance imaging demonstrating myocardial inflammation. Pediatric DCM patient P0003, transplanted at 2.5 years of age, did not have imaging for myocardial inflammation. While medical records for these patients did not suggest myocarditis or notable viral infection, in myocardial sections from each we identified inflammatory infiltrates by immunohistochemistry with an anti-CD68 antibody (Figure 2). Together these data indicate a causal or potential contribution of parvovirus in the pathogenesis of these DCM cases.

### Distribution of genetic variants in end-stage versus ambulatory cardiomyopathy cases

The high number of genetic variants discovered in our WES/WGS analysis of explant cardiac tissue samples from heart transplantation prompted us to compare our findings with recent genetic testing results obtained from non-ischemic cardiomyopathy patients. We reviewed three recent studies, which examined genetic contribution to adult DCM patients regardless of severity of their disease. Verdonschot and colleagues^34^ studied 689 DCM patients using a 48 cardiomyopathy gene panel. We evaluated for pathogenic variants in these 48 genes in our WES/WGS data and noted prevalence of causal variants to be significantly higher in the end-stage DCM cohort (42% versus 19%, p=3.78e-06; Tables 6 and XI). Morales and colleagues^35^ performed focused analysis of 35 cardiomyopathy-associated genes from the WES data of 97 ambulatory DCM patients and identified pathogenic variants in 15% of cases, which is significantly lower than 40% cases in our cohort when comparing the same set of the 35 genes (p=2.24e-04; Tables 6 and XII). In a study by Mazzarotto et al.^6^ the authors recruited 1040 patients with DCM from outpatient clinic and performed genetic evaluation using a gene panel testing including 56 DCM-associated genes. We again noted a significant enrichment of pathogenic variants in patients with end-stage DCM when comparing the same set of the genes (42% versus 18%, p=4.70e-07; Tables 6 and XIII).

The distribution of identified causal genes was similar in published studies and our cohort. TTNtv was the most common disease variant, 51% in pathogenic variant-positive end-stage DCM and 51–64% in pathogenic variant-positive ambulatory DCM cohorts. *LMNA* was the second most common disease gene followed by other DCM-genes such as *BAG3*, *DSP*, *FLNC*, and *RBM20* (Tables XI–XIII).

## Discussion

We demonstrate that genetic analysis of explanted heart tissues was highly informative of the causes for unexplained end-stage cardiomyopathies. Pathogenic variants were identified in 54 of 122 samples (44.3%) including 42.4% in DCM and 50.0% in HCM cases. Additionally, in two tissues we demonstrated 6 to 9-fold enrichment above the cohort mean levels for parvovirus genome sequences and immunohistochemical evidence for active inflammation. With the inclusion of parvovirus infection as the cause for two patients' cardiomyopathies our analysis uncovered genetic and viral etiologies in 56 of 122 patients (45.9%) heart transplant patients.

Recent studies reported genetic diagnostic yield of 15–18% in DCM^6,34,35^ and 27–35% in HCM^13,36^ patients, who underwent gene-panel testing in ambulatory clinic settings. The frequency of damaging variants in DCM patients was significantly higher in our cohort (p<0.001; Table 6). We interpret these findings to indicate that pathogenic variants convey a greater risk for progression to HF than non-genetic causes of DCM. While we analyzed fewer HCM tissues, pathogenic variants were found in 50% of samples. Similar to DCM, these preliminary data indicate that genetic causes of HCM convey substantial risk for progression to end-stage disease. Our conclusions are supported by and extend earlier studies that indicate DCM and HCM patients with pathogenic variants are more likely to develop advanced cardiomyopathy characterized by arrhythmias, NYHA class III/IV, and LVEF < 35%^34,37^.

Prior analyses focused on established or posited cardiomyopathy genes (Table I) that are studied using commercially available cardiomyopathy gene panel tests. When considering these genes, we observed a higher prevalence of causal variants seen in our end-stage cardiomyopathy cohort. Two reasons might account for this observation. First, WES/WGS facilitated the identification of three large genomic structural variants and two noncanonical splicing variants that caused disease in 4% of our study cohort. These types of variants can be missed by traditional gene-panel testing. Second, although American College of Medical Genetics and Genomics (ACMG) and American Heart Association (AHA)/American College of Cardiology (ACC)/Heart Failure Society of America (HFSA) Guideline for the Management of Heart Failure recommended genetic evaluation for patients with non-ischemic cardiomyopathy^23,38^, our findings highlight the even higher value of assessing for pathogenic variants in cardiac transplant patients.

Although a higher fraction of subjects undergoing transplantation have a pathogenic or likely pathogenic variant than other ambulatory DCM cases, the distribution of variants between disease genes was similar in these two cohorts. Notably, all *TTN* variants were found in adult cases while 2 of 7 *LMNA* variants were identified in pediatric DCM cases (Table 2). This data confirms a well-established role of TTNtv in adult-onset DCM, but not in pediatric-onset DCM^9,10^. Rare variants in other established DCM genes including *BAG3*, *DSP*, *TNNT2*, filamin C (*FNLC*), and *MYH7* ranged from 1–3% of cases in our cohort. Statistical comparison of HCM causal variants in our cohort and other cohorts was limited due to the small number of cases in the current study.

We recognize several limitations in this study. The study cohort consists of transplants performed over a 9-year period from a single transplantation center. As pathogenic or likely pathogenic variants were recognized in 43.6% of adults and 42.9% of pediatric samples, the causes of HF for many transplant recipients remain unknown. While our analysis focused on identification of rare genetic variants in protein coding sequences of cardiomyopathy genes, available tissues allowed us to identify and functionally confirmed the effects of one noncoding variant within promoter sequences. Further studies of non-coding sequences may identify pathogenicity of novel genes and may improve the identification of cause in the unsolved cases. Finally, our study was not designed to assess common genetic variants that may modify clinical severity; these may be increased in transplant recipients. As new genetic tools for diagnosing causes of DCM and HCM are identified, genetic data produced here should be re-visited to further define the etiology of end-stage cardiomyopathy.

In summary, our analysis of explanted heart tissues provides unique perspectives in understanding genetic contribution to unexplained end-stage cardiomyopathy. Rare genetic variants contributed to pathogenesis of 42% of end-stage DCM cases and 50% of end-stage HCM irrespective of family history. We propose that comprehensive genetic analyses of explanted heart tissues from transplant patients with unexplained disease should be incorporated into current guidelines. Analyses of these samples improves knowledge about the causes of HF, information that can be used to assess responses to pre-transplant interventions. Additionally these data enable clinical cascade screening of their first-degree relatives per ACMG recommendation^23^ and the opportunities for early treatments to limit the development of HF^39^.

## Supplementary Material

004062 - Supplemental Material

## Figures and Tables

**Table 1.** Clinical characteristics of explant tissues from adult and pediatric heart transplant recipients

	Adult (n=101)				Pediatric (n=21)		
	DCM (n=85)		HCM (n=13)	ACM (n=3)	DCM (n=14)	HCM (n=5)	RCM (n=2)
Clinical							
Age, year	50.0 (39.0–58.0)		45.0 (40.0–55.0)	37.0 (30.0–46.5)	7.4 (2.4–9.3)	2.2 (0.9–7.1)	4.2 (3.9–4.4)
Sex (M / F)	68 / 17		9 / 4	2 / 1	6 / 8	3 / 2	2 / 0
Self-reported race							
White	78 (91.8%)		12 (92.3%)	2 (66.7%)	7 (50.0%)	2 (40.0%)	2 (100.0%)
African American	0 (0.0%)		1 (7.7%)	0 (0.0%)	0 (0.0%)	0 (0.0%)	0 (0.0%)
Asian	6 (7.1%)		0 (0.0%)	1 (33.3%)	3 (21.4%)	2 (40.0%)	0 (0.0%)
Middle Eastern	1 (1.2%)		0 (0.0%)	0 (0.0%)	3 (21.4%)	1 (20.0%)	0 (0.0%)
Comorbidity							
Diabetes	11 (12.9%)	2 (15.4%)		0 (0.0%)	0 (0.0%)	0 (0.0%)	0 (0.0%)
Hypertension	15 (17.6%)	3 (23.1%)		0 (0.0%)	1 (7.1%)	0 (0.0%)	0 (0.0%)
Kidney disease	44 (51.8%)	6 (46.2%)		0 (0.0%)	1 (7.1%)	0 (0.0%)	0 (0.0%)
Pulmonary hypertension	22 (25.9%)	5 (38.5%)		0 (0.0%)	3 (21.4%)	0 (0.0%)	1 (50.0%)
Arrhythmia							
AF/Flutter	26 (30.6%)		6 (46.2%)	2 (66.7%)	1 (7.1%)	0 (0.0%)	0 (0.0%)
VT/VFib	16 (18.8%)		3 (23.1%)	1 (33.3%)	0 (0.0%)	0 (0.0%)	0 (0.0%)
Electrocardiogram							
QRS duration, ms	130.0 (102.0–162.0)		130.0 (102.0–162.0)	130.0 (102.0–162.0)	88.0 (78.0–106.0)	85.0 (77.0–96.3)	89.5 (87.8–91.3)
AF	18 (21.2%)		18 (21.2%)	18 (21.2%)	1 (7.1%)	0 (0.0%)	0 (0.0%)
LBBB	11 (12.9%)		11 (12.9%)	11 (12.9%)	0 (0.0%)	NA	0 (0.0%)
Echocardiography							
LVPWT, mm	8.5 (7.5–10.0)	12.0 (10.3–14.5)		9.6 (9.3–9.8)	5.6 (4.8–6.9)	8.9 (7.5–15.0)	6.1 (4.7–7.6)
LVEF, %	20.0 (13.9–23.7)	27.3 (25.0–46.3)		15.0 (12.6–34.0)	25.3 (17.0–31.3)	72.0 (63.1–83.0)	51.0 (51.0–51.0)
LVIDd, mm	69.0 (60.0–75.0)	46.0 (40.5–55.0)		52.0 (49.0–61.0)	55.0 (44.0–61.0)	24.0 (22.0–29.0)	25.5 (24.3–26.8)
RV systolic dysfunction	4.0 (3.0–4.0)	3.0 (3.0–4.0)		4.0 (4.0–4.0)	2.0 (1.0–2.0)	1.0 (0.0–2.3)	1.0 (0.5–1.5)
Devices							
ICD	47 (55.3%)	12 (92.3%)		3 (100.0%)	1 (7.1%)	1 (20.0%)	0 (0.0%)
BiV-ICD	24 (28.2%)	2 (15.4%)		0 (0.0%)	0 (0.0%)	0 (0.0%)	0 (0.0%)
VAD	55 (64.7%)	6 (46.2%)		0 (0.0%)	8 (57.1%)	1 (20.0%)	0 (0.0%)
Medication							
ACEi/ARB	62 (72.9%)	6 (46.2%)		2 (66.7%)	8 (57.1%)	0 (0.0%)	0 (0.0%)
β-blocker	71 (83.5%)	7 (53.8%)		2 (66.7%)	9 (64.3%)	3 (60.0%)	1 (50.0%)
Amiodarone	40 (47.1%)	6 (46.2%)		1 (33.3%)	1 (7.1%)	0 (0.0%)	0 (0.0%)
MRA	57 (67.1%)	5 (38.5%)		2 (66.7%)	3 (21.4%)	0 (0.0%)	0 (0.0%)

ACEi, angiotensin converting enzyme inhibitors; ACM, arrhythmogenic cardiomyopathy; AF, atrial fibrillation; AF/Flutter, atrial flutter; ARB, angiotensin receptor blockers; BiV-ICD, bi-ventricular implantable cardioverter-defibrillator; CRT, cardiac resynchronization therapy; DCM, dilated cardiomyopathy; Diabetes, type 2 diabetes mellitus; HCM, hypertrophic cardiomyopathy; ICD, implantable cardioverter-defibrillator; LBBB, left bundle-branch block; LVEF, left ventricular ejection fraction; LVIDd, left ventricular internal dimension in diastole; LVPWT, left ventricular posterior wall thickness; MRA, mineralocorticoid receptor antagonists; NA, data not available; RCM, restrictive cardiomyopathy; RV systolic dysfunction, right ventricular systolic dysfunction; VAD, ventricular assist device; VT/VFib, ventricular fibrillation. Categorical variables presented as count with percentage in parenthesis: sex, ethnicity, comorbidity, arrhythmia burden, AF, LBBB, devices, medication. Continuous variables presented as median with interquartile range in parenthesis: age, QRS duration, LVPWT, LVEF, LVIDd. The variables of RV systolic dysfunction were scored as follow: none=0.0, trivial=1.0, mild=2.0, moderate=3.0, severe=4.0

**Table 2.** Distribution of pathogenic cardiomyopathy variants in DCM patients' tissue samples

Gene	Number of variants based on variant type			Number of variants based on age		Total number of variants
	Loss-of-function variants	Protein-altering variants	Structural variants	Adult samples	Pediatric samples	
*TTN*	17	0	1	18	0	18 (18.2%)
*LMNA*	4	3	0	5	2	7 (7.1%)
*BAG3*	2*	0	1	3	0	3 (3.0%)
*DSP*	3	0	0	3	0	3 (3.0%)
*TNNT2*	0	2^†^	0	0	2	2 (2.0%)
*ALMS1*	1^‡^	0	0	0	1	1 (1.0%)
*DMD*	0	0	1	1	0	1 (1.0%)
*FLNC*	1	0	0	0	1	1 (1.0%)
*LAMP2*	1	0	0	1	0	1 (1.0%)
*MYBPC3*	1	0	0	1	0	1 (1.0%)
*MYH7*	0	1	0	1	0	1 (1.0%)
*RBM20*	1	0	0	1	0	1 (1.0%)
*SCN5A*	1	0	0	1	0	1 (1.0%)
*TPM1*	0	1	0	0	1	1 (1.0%)
PVneg	N/A	N/A	N/A	50	7	57 (57.6%)
Total	32	7	3	85	14	99 (100.0%)

* Two individuals from the same family

† P0014 carries *TNNI3* LoF variant in addition to *TNNT2* protein-altering variant

‡ P0007 carries two LoF variants, PVneg: pathogenic variant negative

**Table 3.** Distribution of pathogenic cardiomyopathy variants in HCM patients' tissue samples

Gene	Number of variants based on variant type		Number of variants based on age		Total number of variants
	Loss-of-function variants	Protein-altering variants	Adult samples	Pediatric samples	
*MYBPC3*	3	2	5	0	5 (27.8%)
*MYH7*	N/A	2	2	0	2 (11.1%)
*RIT1*	0	1	0	1	1 (5.5%)
*TNNT2*	0	1	1	0	1 (5.5%)
PVneg	N/A	N/A	5	4	9 (50.0%)
Total	3	6	13	5	18 (100.0%)

PVneg: pathogenic variant negative

**Table 4.** Distribution of pathogenic cardiomyopathy variants in ACM patients' tissue samples

Gene	Number of variants based on variant type		Number of variants based on age		Total number of variants
	Loss-of-function variants	Protein-altering variants	Adult samples	Pediatric samples	
*PKP2*	1	0	1	0	1 (33.3%)
*TMEM43*	0	1	1	0	1 (33.3%)
PVneg	N/A	N/A	1	0	1 (33.3%)
Total	1	1	3	0	3 (100.0%)

PVneg: pathogenic variant negative

**Table 5.** Distribution of pathogenic cardiomyopathy variants in RCM patients' tissue samples

Gene	Number of variants based on variant type		Number of variants based on age		Total
	Loss-of-function variants	Protein-altering variants	Adult samples	Pediatric samples	
*MYH7*	N/A	1	0	1	1 (50.0%)
PVneg	N/A	N/A	0	1	1 (50.0%)
Total	0	1	0	2	2 (100.0%)

PVneg: pathogenic variant negative

**Table 6.** Prevalence of pathogenic variants in end-stage DCM cases compared to DCM cases at all clinical stages

	End-stage DCM (n=85)	All DCM (Study 1, n=689*)	P-val	End-stage DCM (n=85)	All DCM (Study 2, n=97)	P-val	End-stage DCM (n=85)	All DCM (Study 3, n=1040)	P-val
PVpos	36 (42%)	129 (19%)	3.78e-06	34 (40%)	15 (15%)	2.24e-04	36 (42%)	183 (18%)	4.70e-07
PVneg	49 (58%)	560 (81%)		51 (60%)	82 (85%)		49 (58%)	857(82%)	

Data from Verdonschot et al.^34^ (Study 1), Morales et al.^35^ (Study 2), Mazzarotto et al.^6^ (Study 3); P-values calculated using Fisher's exact test

PVneg: pathogenic variant negative, PVpos: pathogenic variant positive

* *FLNC* was tested in 172 cases

## Nonstandard Abbreviations and Acronyms

ACM: arrhythmogenic cardiomyopathy
DCM: dilated cardiomyopathy
HCM: hypertrophic cardiomyopathy
HF: heart failure
LoF: loss-of-function
RCM: restrictive cardiomyopathy
WES: whole exome sequencing
WGS: whole genome sequencing
