# Genetic Contribution to End-Stage Cardiomyopathy Requiring Heart Transplantation

**DOI:** 10.1161/CIRCGEN.123.004062

**Published:** 2023-09-28

**Authors:** Yuri Kim, Oddný Brattberg Gunnarsdóttir, Anissa Viveiros, Daniel Reichart, Daniel Quiat, Jon A.L. Willcox, Hao Zhang, Huachen Chen, Justin J. Curran, Daniel H. Kim, Simon Urschel, Barbara McDonough, Joshua Gorham, Steven R. DePalma, Jonathan G. Seidman, Christine E. Seidman, Gavin Y. Oudit

**Affiliations:** 1 Division of Cardiovascular Medicine, Brigham and Women’s Hospital (Y.K., B.M., C.E.S.).; 2 Department of Genetics, Harvard Medical School, Boston, MA (Y.K., O.B.G., D.R., D.Q., J.A.L.W., J.J.C., J.G., S.R.D., J.G.S., C.E.S.).; 3 Department of Medicine (A.V., H.Z., H.C., D.H.K., G.Y.O.), University of Alberta.; 4 Department of Pediatrics (S.U.), University of Alberta.; 5 Mazankowski Alberta Heart Institute, Edmonton, Canada (A.V., H.Z., H.C., D.H.K., G.Y.O.).; 6 Department of Medicine I, University Hospital, Ludwig Maximilian University of Munich, Germany (D.R.).; 7 Department of Cardiology, Boston Children’s Hospital, MA (D.Q.).; 8 Stollery Children’s Hospital, Edmonton, Alberta, Canada (S.U.).; 9 Howard Hughes Medical Institute, Chevy Chase, MD (B.M., S.R.D., C.E.S.).

**Keywords:** cardiomyopathies, genetics, heart failure, transplantation, whole exome sequencing, whole genome sequencing

## Abstract

**Background:**

Many cardiovascular disorders propel the development of advanced heart failure that necessitates cardiac transplantation. When treatable causes are excluded, studies to define causes are often abandoned, resulting in a diagnosis of end-stage idiopathic cardiomyopathy. We studied whether DNA sequence analyses could identify unrecognized causes of end-stage nonischemic cardiomyopathy requiring heart transplantation and whether the prevalence of genetic causes differed from ambulatory cardiomyopathy cases.

**Methods:**

We performed whole exome and genome sequencing of 122 explanted hearts from 101 adult and 21 pediatric patients with idiopathic cardiomyopathy from a single center. Data were analyzed for pathogenic/likely pathogenic variants in nuclear and mitochondrial genomes and assessed for nonhuman microbial sequences. The frequency of damaging genetic variants was compared among cardiomyopathy cohorts with different clinical severity.

**Results:**

Fifty-four samples (44.3%) had pathogenic/likely pathogenic cardiomyopathy gene variants. The frequency of pathogenic variants was similar in pediatric (42.9%) and adult (43.6%) samples, but the distribution of mutated genes differed (*P*=8.30×10^-4^). The prevalence of causal genetic variants was significantly higher in end-stage than in previously reported ambulatory adult dilated cardiomyopathy cases (*P*<0.001). Among remaining samples with unexplained causes, no damaging mitochondrial variants were identified, but 28 samples contained parvovirus genome sequences, including 2 samples with 6- to 9-fold higher levels than the overall mean levels in other samples.

**Conclusions:**

Pathogenic variants and viral myocarditis were identified in 45.9% of patients with unexplained end-stage cardiomyopathy. Damaging gene variants are significantly more frequent among transplant compared with patients with ambulatory cardiomyopathy. Genetic analyses can help define cause of end-stage cardiomyopathy to guide management and risk stratification of patients and family members.

Heart failure (HF) is a devastating disorder with high morbidity and mortality, affecting at least 26 million people worldwide^1^ and leading to death in 50% of patients within 5 years after the first HF hospitalization.^2^ Cardiomyopathy, a pathological remodeling of the myocardium that precedes HF, is typically classified as ischemic or nonischemic origin.

Genetic causes of nonischemic cardiomyopathy are increasingly recognized.^3,4^ Dilated (DCM), hypertrophic (HCM), and arrhythmogenic (ACM) cardiomyopathies are often monogenic disorders caused by damaging variants within protein-coding sequences or arise from polygenic risk variants^3^ and environmental factors.^5^ A causal genetic variant is found in ≈10% to 30% of sporadic DCM and 30% to 50% of HCM cases.^6,7^ Genetic causes of DCM are more varied and scores of putative DCM genes have been hypothesized. The putative DCM genes are involved in various cellular functions within cardiomyocytes including contractile apparatus (sarcomeres), calcium cycling, nuclear membranes, heat shock chaperones, mitochondria, and cell adhesion.^8^ Pathogenic variants in gene titin (*TTN*) are the most common genetic cause of adult-onset DCM, found in 15% to 20% of affected individuals, but rarely identified in childhood-onset DCM.^9,10^ TTN serves as a scaffold to support formation of the contractile apparatus and a molecular spring that influences contractility and relaxation. Genetic variants in *TTN* that cause DCM by creating premature truncation of the protein are denoted TTNtv.^11^ The second most common genetic cause of DCM is rare pathogenic variants in lamin A/C (*LMNA*), which encodes a ubiquitously expressed nuclear membrane protein that participates in the maintenance of nuclear structure. *LMNA* variants account for ≈5% of DCM cases and have a higher prevalence among patients with DCM with cardiac conduction abnormalities.^12^

Pathogenic variants that cause HCM generally occur in 8 sarcomere genes encoding: myosin binding protein C (*MYBPC3*), myosin heavy chain (*MYH7*), regulatory myosin light chain (*MYL2*), essential myosin light chain (*MYL3*), troponin I (*TNNI3*), troponin T, (*TNNT2*), tropomyosin (*TPM1*), and alpha cardiac actin (*ACTC1*).^7,13^ Pathogenic variants in *MYBPC3* and *MYH7* predominate, accounting for up to 50% of all HCM cases and 75% of cases with a defined genetic cause.^13^ About 3% to 8% of HCM patients develop end-stage HCM characterized by left ventricular ejection fraction <50%. Approximately 30% to 50% of end-stage HCM patients develop refractory HF and 11% to 25% require heart transplant.^14^

ACM is characterized by myocardial atrophy and fibrofatty replacement of the myocardium that may predominately alter the right ventricle or cause biventricular dysfunction.^15^ The prevalence of ACM is estimated to be 1:2000 to 1:5000 and typically follows an autosomal dominant inheritance pattern^16^ albeit with variable penetrance. Pathogenic variants in desmosome genes are the most common genetic variants in ACM and include plakophilin 2 (*PKP2*), desmoplakin (*DSP*), desmocollin 2 (*DSC2*), junction plakoglobin (*JUP*), and desmoglein 2 (*DSG2*).^17^

Cardiomyopathies that arise during childhood and cause HF are rare and often lethal conditions.^18^ The causes of cardiomyopathy in children are more diverse than in adults and include metabolic, neuromuscular, and syndromic genes.^19^ Additionally, pathogenic variants in the mitochondrial genome, which occur in 1 in 5000 newborns cause cardiomyopathies in 40% of cases, with high rates of early mortality.^20^ As children with mitochondrial cardiomyopathies have greater rates of morbidities after transplantation, careful selection of appropriate candidates has been urged.^21^ Acute myocarditis is a rare cause of childhood HF, but longitudinal studies indicate persistent myocardial dysfunction in almost 50% of cases, with almost 19% requiring cardiac transplantation.^22^

Despite the recognition of heritable^23^ and microbial^24^ causes of cardiomyopathies, little is known about the contribution of these to end-stage HF requiring transplant. A previous study from Spain reported causal genetic variants in 73% of 52 transplant patients with familial DCM, including 25% with a founder mutation in the emerin (*EMD*) gene.^25^ Another study employed gene panel testing of peripheral blood from 31 heart transplant patients with nonischemic cardiomyopathy and identified pathogenic and likely pathogenic variants in 38.7%.^26^

Comprehensive cardiac tissue DNA analyses to interrogate germline nuclear, mitochondrial, and microbial sequences provide the opportunity to define causes of heretofore idiopathic HF. Therefore, we analyzed whole-exome sequences (WES) and whole genome sequences (WGS) sequences of explanted nonischemic cardiomyopathy tissues from heart transplants, regardless of family history of cardiomyopathy, to identify and define the prevalence of pathogenic variants in end-stage cardiomyopathy.

## METHODS

This study was performed using de-identified codes and protocols that were reviewed and approved by the human research committees at University of Alberta (Edmonton, Canada) and Brigham and Women’s Hospital at Harvard Medical School (Boston). All patients or their family provided written informed consent. The data and analytic methods will be made available to other researchers on request. Further details are provided in Supplemental Methods.

## RESULTS

### Clinical Characteristics of Study Population

We performed WES/WGS analyses on 122 explant tissues from patients with idiopathic cardiomyopathies undergoing orthotopic heart transplantation (Table 1). No patient had prior genetic analyses. Clinical diagnoses in 101 adult patients included DCM (n=85), HCM (n=13), and ACM (n=3). Twenty-one pediatric patients were diagnosed with DCM (n=14), HCM (n=5), and restrictive cardiomyopathy (RCM; n=2). Adult patients were predominantly male (78.2%), self-identified as white (91.1%), and had mean ages at transplant of 50 years (DCM), 45 years (HCM), and 37 years (ACM). Two patients with DCM were related. Pediatric cases were 47.6% females, self-reported as nonwhite (42.9%), and had mean ages at transplant of 7.4 years (DCM), 2.2 years (HCM), and 4.2 years (RCM).

**Table 1.**
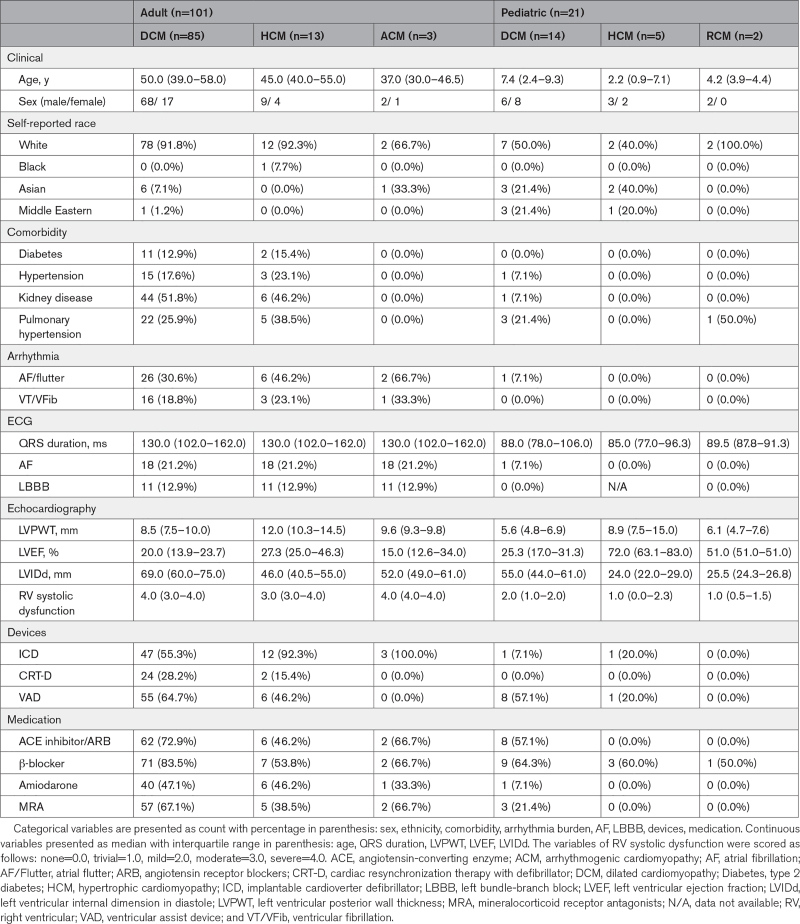
Clinical Characteristics of Explant Tissues From Adult and Pediatric Heart Transplant Recipients

### Rare Pathogenic Variants in DCM Explanted Hearts

To determine contribution of genetic variants to end-stage DCM, we performed WES/WGS analysis of 99 explant DCM tissues from heart transplantation. From initial analyses of coding variants, we identified 42 pathogenic and likely pathogenic variants in 53 cardiomyopathy genes (Table S1). These included 32 loss-of-function (LoF) variants, 7 missense variants, and 3 samples with large structural variants, each causing autosomal dominant disease (Table 2; Tables S3 through S5). Damaging variants in TTN accounted for 42.9% of genotype-positive DCM cases, followed by *LMNA* (16.7%), Bcl-2 associated athanogene 3 (*BAG3*; 7.1%), and *DSP* (7.1%; Table 2; Tables S3 through S5). Two related individuals shared the same LoF variant in BAG cochaperone 3 (BAG3 p.Arg123*; Table S3). We also identified 1 rare splicing and 6 rare damaging missense variants in posited DCM genes (Tables S2 and S7) with uncertain pathogenicity (Table 3).

**Table 2.**
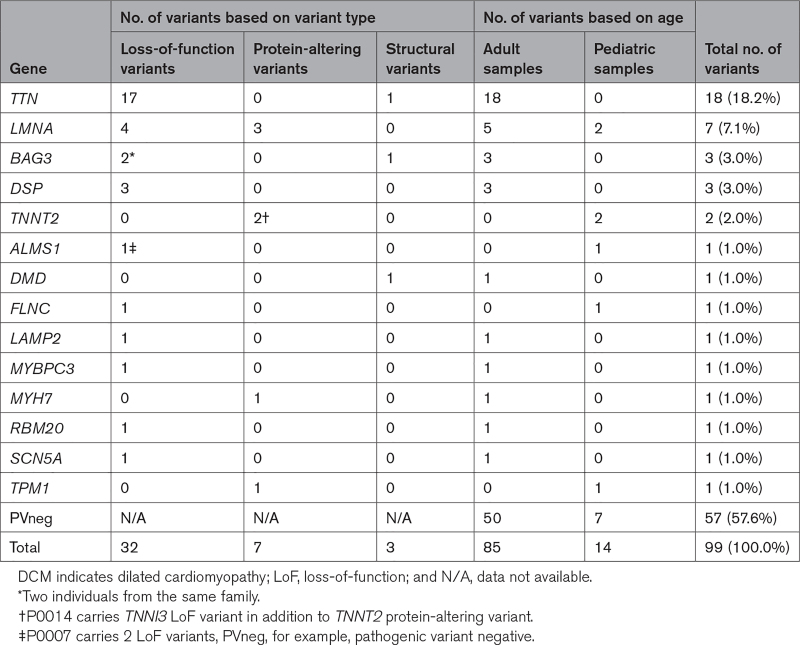
Distribution of Pathogenic Cardiomyopathy Variants in Tissue Samples of Patients With DCM

**Table 3.**
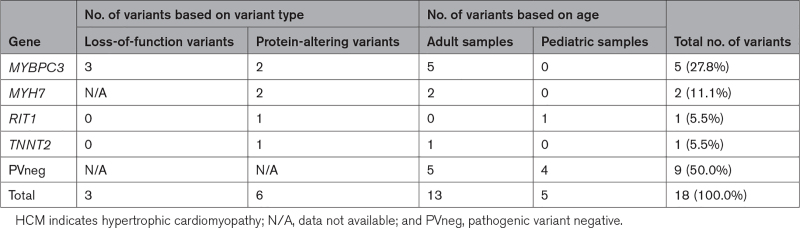
Distribution of Pathogenic Cardiomyopathy Variants in Tissue Samples of Patients With HCM

**Figure 1.**
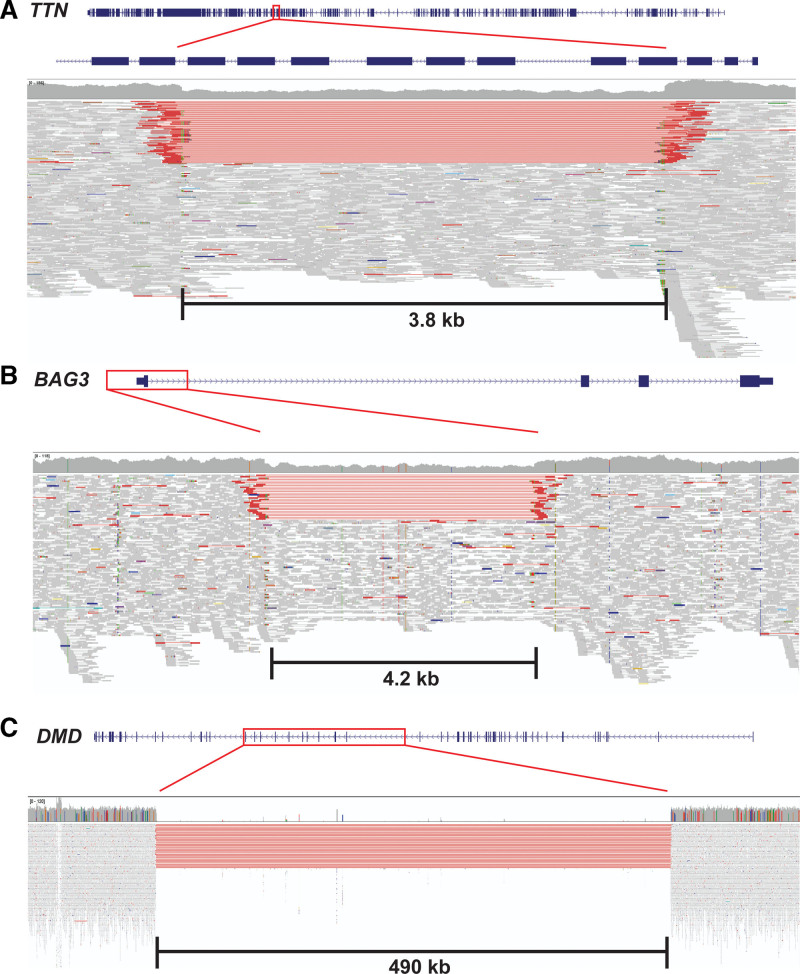
**Large structural variants were identified in dilated cardiomyopathy (DCM) cases. A**, A 3.8 kb deletion in *TTN* involving seven exons leading to out-of-frame deletion. **B**, A 4.2 kb deletion involving the promoter and exon 1 of *BAG3*, leading to haploinsufficiency. **C**, A 490 kb deletion leading to hemizygous in-frame deletion of *DMD* in a male patient. Figures are modified from University of California Santa Cruz (UCSC) Genome Browser^28^ and Integrative Genomics Viewer.^29^ Red box in each part indicates location of the structural variant relative to each affected gene. Height of vertical thick gray bars in the **middle** of each part indicates the number of sequence reads mapping to the reference genome at a particular location. Thin horizontal gray lines at the **bottom** of each part represent sequencing reads aligned to sequences of each gene. Thin horizontal red lines demonstrate sequencing reads that span over a long distance within the genome, indicating the presence of large sequence deletions.

Several genes had atypical types of pathogenic variants. A structural variant in *TTN* (sample A0036) removes 7 exons with high proportion spliced in^27^ values and creates an out-of-frame deletion (Figure 1A). A structural variant in *BAG3* (sample A0097) leads to deletion of the promoter and exon 1 of *BAG3* resulting in haploinsufficiency (Figure 1B), which was confirmed via RNA-sequencing analysis. Sample A0187’s genome contains a large structural variant in dystrophin (*DMD*), which is predicted to encode a large in-frame deletion of DMD (Figure 1C). RNA-sequencing analyses of A0187 RNA confirmed the multiexon loss in DMD RNA. Overall, next-generation sequencing analysis enabled genetic diagnosis in 42 of 99 end-stage DCM cases (42.4%).

Genetic data altered 3 patients’ clinical diagnosis of DCM. Patient A0127 carried a prototypical pathogenic HCM mutation (*MYBPC3* c.26-2A>G). The male patient (A0187) with an in-frame *DMD* had a history of chronically elevated creatinine kinase with negative evaluations for autoimmune myopathy/myositis and was reclassified with Becker muscular dystrophy. Female patient A0035 carried a heterozygous splice-site variant in lysosome-associated membrane protein 2 (*LAMP2*) that encoded a deletion and frameshift. Although males with LoF variants in X-linked *LAMP2* have Danon disease with massive cardiac hypertrophy, prevalent arrhythmias, and skeletal muscle, hepatic, and neurocognitive dysfunction, she like other women with damaging *LAMP2* variants^30^ had no extracardiac manifestations.

### Rare Pathogenic Variants in HCM, ACM, and RCM Explanted Hearts

Genetic analyses of 18 tissues from patients with clinical diagnosis of HCM identified causal variants in 50% of samples, including 3 LoF and 6 damaging missense variants (Table 3; Table S8). Among 13 adult patients 8 pathogenic variants were identified (61.5%) in sarcomere genes *MYBPC3* (5 variants), *MYH7* (2 variants), and *TNNT2* (1 variant). In contrast, only 1 of 5 pediatric HCM samples (20.0%) had a damaging variant, in a nonsarcomere gene *RIT1* (encoding Ras-like without CAAX protein-1). Activating *RIT1* variants cause Noonan syndrome, a finding that supports distinct genetic causes and molecular pathophysiology in pediatric- and adult-onset HCM.^31^

Among the few patients with ACM (n=3) and RCM (n=2), 3 causal genetic variants were identified. ACM variants included a LoF variant in *PKP2* and a missense founder variant TMEM43 (transmembrane protein 43) p.Ser358Leu, predicted to have arisen in Europe 1300 to 1500 years ago and recurrently observed in Canadian (Newfoundland) subjects^32^ (Table 4; Table S9). Both RCM cases were from pediatric patients, and a damaging missense variant in *MYH7* was found in 1 sample (Tables 5; Table 10).

**Table 4.**
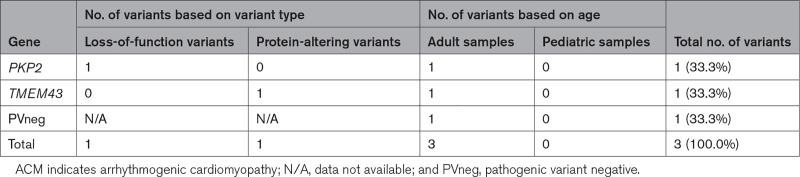
Distribution of Pathogenic Cardiomyopathy Variants in Tissue Samples of Patients With ACM

**Table 5.**
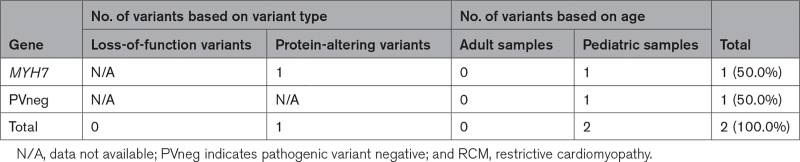
Distribution of Pathogenic Cardiomyopathy Variants in Tissue Samples of Patients With RCM

### Analyses of Mitochondrial Variants and Microbial Sequences in Explanted Hearts

For 86 explanted tissues from 65 adult and 21 pediatric patients with unsolved cardiomyopathies after exome analyses, we examined mitochondrial sequences within WGS data. No pathogenic variants were identified with a heteroplasmic fraction^33^ >0.1. Additionally, among patients of European ancestry, there was no mitochondrial haplotype enrichment compared to ancestry-matched controls.

**Figure 2.**
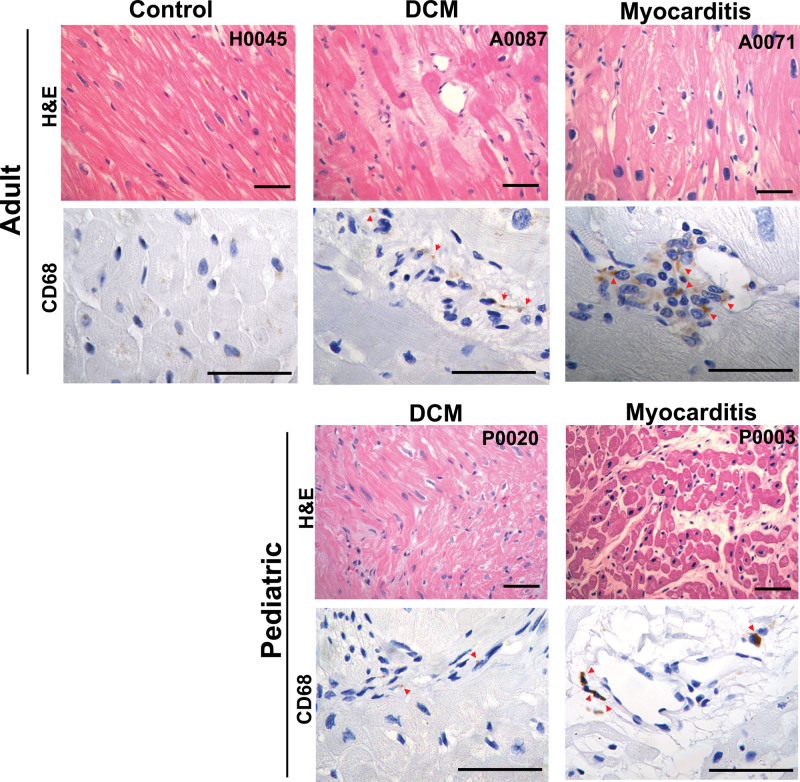
**Myocardial histological staining of adult and pediatric cases with parvovirus infection.** Histology of the human heart using hematoxylin and eosin staining (**top**), and immunohistochemical (IHC) staining for CD68 (cluster of differentiation 68)-positive macrophages (**bottom**). Staining was performed for control, dilated cardiomyopathy (DCM), and potential myocarditis cases from adult and pediatric donors (scale bar: 50 µm). Red arrows point to positive staining in IHC images.

We then examined WGS for the presence of genomic sequences of parvovirus B19, the most common cause of viral myocarditis.^24^ Twenty-eight of 86 tissues carried sequences for parvovirus B19. Across all samples, the read counts encoding parvovirus B19 ranged from 2 to 197 (mean, 21.9; median, 9). Explanted tissues from 2 patients had strikingly higher viral read counts: 125 (A0071) and 197 (P0003). Patient A0071, an adult with DCM, had cardiac magnetic resonance imaging demonstrating myocardial inflammation. Pediatric DCM patient P0003, transplanted at 2.5 years of age, did not have imaging for myocardial inflammation. While medical records for these patients did not suggest myocarditis or notable viral infection, in myocardial sections from each we identified inflammatory infiltrates by immunohistochemistry with an anti-CD68 (cluster of differentiation 68) antibody (Figure 2). Together these data indicate a causal or potential contribution of parvovirus in the pathogenesis of these DCM cases.

### Distribution of Genetic Variants in End-Stage Versus Ambulatory Cardiomyopathy Cases

The high number of genetic variants discovered in our WES/WGS analysis of explant cardiac tissue samples from heart transplantation prompted us to compare our findings with recent genetic testing results obtained from nonischemic cardiomyopathy patients. We reviewed 3 recent studies, that examined genetic contribution to adult patients with DCM regardless of severity of their disease. Verdonschot and colleagues^34^ studied 689 patients with DCM using a 48 cardiomyopathy gene panel. We evaluated pathogenic variants in these 48 genes in our WES/WGS data and noted prevalence of causal variants to be significantly higher in the end-stage DCM cohort (42% versus 19%, *P*=3.78×10^−6^; Table 6; Table S11). Morales et al^35^ performed focused analysis of 35 cardiomyopathy-associated genes from the WES data of 97 ambulatory patients with DCM and identified pathogenic variants in 15% of cases, which is significantly lower than 40% of cases in our cohort when comparing the same set of the 35 genes (*P*=2.24×10^−4^; Table 6; Table S12). In a study by Mazzarotto et al,^6^ the authors recruited 1040 patients with DCM from outpatient clinic and performed genetic evaluation using a gene panel testing including 56 DCM-associated genes. We again noted a significant enrichment of pathogenic variants in patients with end-stage DCM when comparing the same set of the genes (42% versus 18%, *P*=4.70×10^−7^; Table 6; Table S13).

**Table 6.**
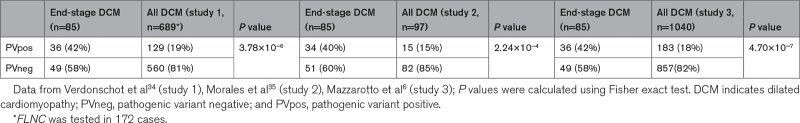
Prevalence of Pathogenic Variants in End-Stage DCM Cases Compared With DCM Cases at All Clinical Stages

The distribution of identified causal genes was similar in published studies and our cohort. TTNtv was the most common disease variant, with 51% in pathogenic variant–positive end-stage DCM and 51% to 64% in pathogenic variant-positive ambulatory DCM cohorts. *LMNA* was the second most common disease gene followed by other DCM genes, such as *BAG3*, *DSP*, *FLNC*, and *RBM20* (Table S11 through S13).

## DISCUSSION

We demonstrate that genetic analysis of explanted heart tissues was highly informative of the causes of unexplained end-stage cardiomyopathies. Pathogenic variants were identified in 54 of 122 samples (44.3%) including 42.4% in DCM and 50.0% in HCM cases. Additionally, in 2 tissues we demonstrated 6- to 9-fold enrichment above the cohort mean levels for parvovirus genome sequences and immunohistochemical evidence for active inflammation. With the inclusion of parvovirus infection as the cause for 2 patients’ cardiomyopathies, our analysis uncovered genetic and viral causes in 56 of 122 patients (45.9%) heart transplant patients.

Recent studies reported genetic diagnostic yield of 15% to 18% in DCM^6,34,35^ and 27% to 35% in HCM^13,36^ patients, who underwent gene panel testing in ambulatory clinic settings. The frequency of damaging variants in patients with DCM was significantly higher in our cohort (*P*<0.001; Table 6). We interpret these findings to indicate that pathogenic variants convey a greater risk for progression to HF than nongenetic causes of DCM. While we analyzed fewer HCM tissues, pathogenic variants were found in 50% of samples. Similar to DCM, these preliminary data indicate that genetic causes of HCM convey substantial risk for progression to end-stage disease. Our conclusions are supported by and extend earlier studies that indicate DCM and HCM patients with pathogenic variants are more likely to develop advanced cardiomyopathy characterized by arrhythmias, New York Heart Association (NYHA) class III/IV, and left ventricular ejection fraction <35%.^34,37^

Prior analyses focused on established or posited cardiomyopathy genes (Table I) that are studied using commercially available cardiomyopathy gene panel tests. When considering these genes, we observed a higher prevalence of causal variants seen in our end-stage cardiomyopathy cohort. Two reasons might account for this observation. First, WES/WGS facilitated the identification of 3 large genomic structural variants and 2 noncanonical splicing variants that caused disease in 4% of our study cohort. These types of variants can be missed by traditional gene panel testing. Second, although American College of Medical Genetics and Genomics and American Heart Association/American College of Cardiology/Heart Failure Society of America Guideline for the Management of Heart Failure recommended genetic evaluation for patients with nonischemic cardiomyopathy,^23,38^ our findings highlight the even higher value of assessing for pathogenic variants in cardiac transplant patients.

Although a higher fraction of subjects undergoing transplantation have a pathogenic or likely pathogenic variant than other ambulatory DCM cases, the distribution of variants between disease genes was similar in these 2 cohorts. Notably, all *TTN* variants were found in adult cases while 2 of 7 *LMNA* variants were identified in pediatric DCM cases (Table 2). This data confirms a well-established role of TTNtv in adult-onset DCM, but not in pediatric-onset DCM.^9,10^ Rare variants in other established DCM genes including *BAG3*, *DSP*, *TNNT2*, filamin C (*FNLC*), and *MYH7* ranged from 1% to 3% of cases in our cohort. Statistical comparison of HCM causal variants in our cohort and other cohorts was limited due to the small number of cases in the current study.

We recognize several limitations in this study. The study cohort consists of transplants performed over a 9-year period from a single transplantation center. As pathogenic or likely pathogenic variants were recognized in 43.6% of adults and 42.9% of pediatric samples, the causes of HF for many transplant recipients remain unknown. Although our analysis focused on identification of rare genetic variants in protein-coding sequences of cardiomyopathy genes, available tissues allowed us to identify and functionally confirm the effects of 1 noncoding variant within promoter sequences. Further studies of noncoding sequences may identify the pathogenicity of novel genes and may improve the identification of cause in the unsolved cases. Finally, our study was not designed to assess common genetic variants that may modify clinical severity; these may be increased in transplant recipients. As new genetic tools for diagnosing causes of DCM and HCM are identified, genetic data produced here should be revisited to further define the cause of end-stage cardiomyopathy.

In summary, our analysis of explanted heart tissues provides unique perspectives in understanding genetic contribution to unexplained end-stage cardiomyopathy. Rare genetic variants contributed to pathogenesis of 42% of end-stage DCM cases and 50% of end-stage HCM irrespective of family history. We propose that comprehensive genetic analyses of explanted heart tissues from transplant patients with unexplained diseases should be incorporated into current guidelines. Analyses of these samples improve knowledge about the causes of HF, information that can be used to assess responses to pretransplant interventions. Additionally, these data enable clinical cascade screening of their first-degree relatives per American College of Medical Genetics and Genomics recommendation^23^ and the opportunities for early treatments to limit the development of HF.^39^

## ARTICLE INFORMATION

### Acknowledgments

The authors thank the patients and families, who participated in this study.

### Sources of Funding

This study was supported by National Institutes of Health (TR002542 to Y. Kim, HL080494 to J.G. Seidman and C.E. Seidman, HL084553 to J.G. Seidman), Brigham and Women’s Hospital (Khoury Innovation Award to Y. Kim), the Howard Hughes Medical Institute (to C.E. Seidman), University Hospital Foundation (to G.Y. Oudit), and Canadian Institutes of Health Research (PJT-425751 to G.Y. Oudit).

### Disclosures

J.G. Seidman and C.E. Seidman are founders of Myokardia (a Bristol-Myers-Squibb Subsidiary) and Consultants for Maze and BridgeBio. C.E. Seidman serves on the Merck Board of Directors. The other authors report no conflicts.

### Supplemental Material

Supplemental Methods

Tables S1–S13

References 6, 28, 29, 33–35, 40–50

## Supplementary Material
