# Supplementary material for: Genetic Contribution to End-Stage Cardiomyopathy Requiring Heart Transplantation
Source: Circ Genom Precis Med. 2023 Sep 28;16(5):452–61. doi: 10.1161/CIRCGEN.123.004062 (PMC10715239; doi:10.1161/CIRCGEN.123.004062)
Supplement: Supplementary file 1 [file hcg-16-452-s001.pdf]

SUPPLEMENTAL MATERIAL

Table of Contents

Supplemental Methods

Supplemental Table I. Definite/likely definite cardiomyopathy-associated genes (n=53)

Supplemental Table II. DCM-associated genes with moderate and limited evidence (n=21)

Supplemental Table III. Loss-of-function variants in definite cardiomyopathy-associated genes in 99 DCM samples

Supplemental Table IV. Damaging-missense variants in definite cardiomyopathy-associated genes in 99 DCM samples

Supplemental Table V. Structural variants in definite cardiomyopathy-associated genes in 99 DCM samples

Supplemental Table VI. Splicing variants in definite cardiomyopathy-associated genes in 99 DCM samples

Supplemental Table VII. Rare loss-of-function, damaging-missense, and splicing variants in DCM-associated genes with moderate and limited evidence found in explant DCM tissues

Supplemental Table VIII. Pathogenic variants in definite cardiomyopathy-associated genes in 18 HCM samples

Supplemental Table IX. Pathogenic variants in definite cardiomyopathy-associated genes in 3 ACM samples

Supplemental Table X. Pathogenic variants in definite cardiomyopathy-associated genes in 2 RCM samples

Supplemental Table XI. The number of pathogenic variants 48 genes tested in Verdonschot et al.[34]

Supplemental Table XII. The number of pathogenic variants 35 genes tested in Morales et al.[35]

Supplemental Table XIII. The number of pathogenic variants 57 genes tested in Mazzarotto et al.[6]

References [6, 28, 29, 33-35, 40-50]

Supplemental Methods

Study population

A total of 213 adult and 55 pediatric heart transplantations were performed at University of Alberta (Edmonton, Canada) during 5/2011-11/2019. Seven adult patients declined to participate in the study. The remaining 206 adult cases consisted of 101 non-ischemic cardiomyopathy, 76 ischemic cardiomyopathy, 13 congenital heart disease, 7 cardiac allograft vasculopathy, 4 valvular disease, 3 sarcoidosis, 1 rheumatic heart disease and 1 eosinophilic myocarditis. Fifty-five pediatric cases comprise 31 congenital heart disease, 21 non-ischemic cardiomyopathy, and 3 cardiac allograft vasculopathy.

Adult and pediatric patients with idiopathic non-ischemic cardiomyopathy were eligible to participate in the current study, irrespective of gender, race, or ethnicity. The tissues were collected from total 122 explanted hearts (101 adult and 21 pediatric) via the Human Explanted Heart Program (HELP). These carried clinical diagnosis of DCM (85 adult and 14 pediatric samples), HCM (13 adult and 5 pediatric), ACM (3 adult), and restrictive cardiomyopathy (RCM; 2 pediatric). This study was performed using protocols that were reviewed and approved by the human research committees at University of Alberta and Brigham and Women's Hospital at Harvard Medical School (Boston, USA). All patients and/or their family provided written informed consent. Left ventricular tissues were obtained at the time of cardiac transplantation and labelled with de-identified codes. Similarly, clinical information was provided to the research laboratory using de-identified codes.

Next generation sequencing and variant analysis

For WES analysis, exome enrichment of genomic DNA extracted from tissue samples was performed with IDT xGen Human Exome Research Panel Version 1.0 and sequenced at the Yale Center for Genome Analysis on an Illumina NovaSeq instrument as 101-base paired-end reads. Reads were mapped to the human reference genome hg19/GRCh37 using the BWA-MEM aligner[40]. For WGS analysis, genomic DNA extracted from tissue samples was sequenced using Illumina HiSeq instruments. All sequencing reads were aligned to hg38 (GRCh38) using BWA-MEM with the -Y option (BWA v0.7.15).

Single nucleotide variants (SNVs) and small indels were identified using the Genome Analysis Tool Kit (GATK) Haplotype Caller tool[41] and filtered for high quality variants. The SNVs were annotated using vcfanno, dbSNP, gnomAD, SnpEff[42], and dbNSFP[43]. High-quality variants (pass GATK Variant Score Quality Recalibration (VSQR) truth sensitivity threshold 99.5 for SNVs and 99.0 for indels, a minimum depth of 10, genotype quality ≥ 20, and Phred-scaled quality score (QUAL) ≥ 30) were filtered for rare (defined as minor allele frequency (MAF) < 1.00e-04 in gnomAD[44]) heterozygous variants. Loss-of-function variants (labeled by SnpEff as nonsense, canonical splice-site, frameshift indels, start loss, stop lost, and stop gained) and protein-altering variants with pathogenic or likely pathogenic annotation in ClinVar[45] in cardiomyopathy-associated genes (Table I) were considered pathogenic to the disease. Splicing variants were identified using SpliceAI[46] and confirmed via RNA-seq analysis.

WGS structural variants were genotyped using a GATK-SV workflow[47] in Terra. Briefly, initial structural variant evidence was collected using the tools Manta, WHAM, GATK-gCNV, cnMOPs, and MELT. SV evidence was then merged across all samples and joint genotyping of SVs across the entire cohort was performed by GATK-SV. Predicted functional effects of genomic SVs were annotated within the GATK-SV pipeline. LoF SVs were defined as deletions overlapping at least a single exon, insertions with exons, inversions with a single or both breakpoints within a gene, and duplications with both breakpoints within exons. To maximize sensitivity, the unfiltered SV call set was evaluated for SVs predicted to result in loss of function.

For variant analysis of DCM genes with limited and moderate evidence (Table II), we filtered for rare (MAF < 1e-04) loss-of-function, damaging missense and splicing variants predicted by in silico algorithms MetaSVM[48] and SpliceAI[46].

Analysis of mitochondrial and parvovirus genomic sequences in explant tissue samples

Mitochondrial variant analysis was performed as previously described[33]. Briefly, short DNA sequence reads from WGS were first aligned to GRCh38 with BWA-MEM[40], chrM reads were extracted and subsequently aligned to the RSRS2 mitochondrial reference sequence using the MToolBox[49]

Parvovirus genome sequences were assessed by aligning non-aligned short read DNA sequences from WGS to all available parvovirus B19 sequences (NCBI Virus database; https://www.ncbi.nlm.nih.gov/labs/virus/vssi/#/virus?SeqType_s=Nucleotide&VirusLineage_ss=Human%20parvovirus%20B19,%20taxid:10798). Reads were considered positive for parvovirus if both reads of a pair of Illumina reads were more than 95% homologous to parvovirus.

RNA sequencing (RNA-seq) analysis

RNA was extracted using TRIzol (Thermo Fisher Scientific) and cDNA was generated using SuperScript III First Strand Synthesis System (Thermo Fisher Scientific). cDNA products were used to construct libraries with the Nextera XT DNA Sample Preparation Kit (Illumina), which were subsequently sequenced with a read length of 75 bp on Illumina NextSeq 500. Reads were aligned to hg38/GRCh38 reference genome using STAR[50]. The aligned sequencing reads were visualized using UCSC Genome Browser[28] and Integrative Genomics Viewer[29] to confirm the presence or absence of RNA encoding structural variations detected in genomic DNA.

Hematoxylin and Eosin (H&E) staining

Formalin-fixed paraffin embedded tissues were sectioned onto slides at 5 μm thickness. Slides were de-waxed and brought to distilled water by alcohol gradient. Nuclei were stained with Harris Hematoxylin for 15 seconds, rinsed, and differentiated with 1% acid alcohol. Bluing was achieved by rinsing in Scott's tap water substitute (20mM Sodium Bicarbonate, 166mM Magnesium Sulfate), then slides were stained with Eosin for two minutes. Slides were dehydrated, cleared and mounted. Imaging was performed with a Leica DM4000 B LED microscope system.

Immunohistochemical staining (IHC)

Immunohistochemical staining was performed for CD68 as a macrophage marker. Briefly, formalin-fixed paraffin embedded tissues were sectioned onto slides at 5μm thickness, de-waxed, and rehydrated by washing stepwise with decreasing concentrations of ethanol. Heat induced epitope retrieval was performed with sodium citrate buffer (10mM Sodium Citrate, 0.05% Tween 20, pH 6.0). Slides were blocked with 10% goat serum in 1% bovine serum albumin (BSA) and incubated with CD68 primary antibody diluted (1:100) in 1% BSA (MA5-13324; Thermo Fisher Scientific) overnight at 4°C. Endogenous peroxidases were blocked with 10% H_2O_2 for 10 minutes, then slides were incubated with HRP-conjugated secondary antibodies (#7076, Cell Signaling Technology) at 1:1000 dilution for 1 hour at room temperature. Positive staining was visualized with freshly prepared DAB (3,3'Diaminobenzidine) substrate (Abcam, Cambridge, United Kingdom) for 10 minutes. Slides were counterstained with regressive Harris Hematoxylin for 15 seconds and differentiated with 1% acid alcohol. Slides were dehydrated by alcohol gradient, cleared, and mounted with organic media. Imaging was performed with a Leica DM4000 B LED microscope system.

Analysis of pathogenetic variants in end-stage versus ambulatory DCM cases

Prevalence of pathogenic variants in adult DCM cases at all clinical stages was obtained from three recently published studies[6,34,35], which provided the list of cardiomyopathy-associated genes evaluated, the number of cases with pathogenic variants for each gene tested or the list of identified rare variants, and the total number of cases examined in the study. We reviewed pathogenic variants in the genes studied in each of recent publication in the WES/WGS data of our end-stage DCM cohort to perform comparison of the same set of genes. Criteria for pathogenicity included rare (MAF < 1.00e-04 in gnomAD[44]), loss-of-function (labeled by SnpEff as nonsense, canonical splice-site, frameshift indels, start loss, stop lost, and stop gained), and protein-altering variants with pathogenic or likely pathogenic annotation in ClinVar[45]. Then, we assessed prevalence of causal variants and performed Fisher's exact test to compare prevalence of causal variants in end-stage versus ambulatory cardiomyopathy cases (Tables 3 and XI-XIII).

Supplemental Table I. Definite/likely definite cardiomyopathy-associated genes (n=53)

ABCC9	*DES*	*HRAS**	*MYL2*	*RIT1**	*TMEM43*
ACTC1	*DMD*	*JUP*	*MYL3*	*RYR2*	*TNNC1*
ACTN2	*DOLK*	*KRAS**	*NRAS**	*SCN5A*	*TNNI3*
ALMS1	*DSC2*	*LAMP2*	*PKP2*	*SGCD*	*TNNT2*
ALPK3	*DSG2*	*LMNA*	*PLN*	*SHOC2**	*TPM1*
BAG3	*DSP*	*MAP2K1**	*PRKAG2**	*SOS1**	*TTN*†
*BRAF**	*EMD*	*MAP2K2**	*PTPN11**	*SOS2**	*TTR*
CRYAB	*FLNC*	*MYBPC3*	*RAF1**	*TAZ*	*VCL*
CSRP3	*GLA*	*MYH7**	*RBM20*	*TCAP*	

*Only damaging-missense variants are considered pathogenic
†Only loss-of-function variants are considered pathogenic

Supplemental Table II. DCM-associated genes with moderate and limited evidence (n=21)

ANKRD1	*CTF1*	*DTNA*	*EYA4*	*GATAD1*	*ILK*
JPH2	*LAMA4*	*LDB3*	*MYH6*	*MYL2*	*MYPN*
NEBL	*NEXN*	*NKX2-5*	*OBSCN*	*PLEKHM2*	*PRDM16*
PSEN2	*TBX20*	*TNNI3K*			

Supplemental Table III. Loss-of-function variants in definite cardiomyopathy-associated genes in 99 DCM samples

ID	Gene	GRCh38 coordinate	CDS change	AA change	ClinVar
A0053	*ALMS1*[*]	chr2:73452450GA>G	c.5924delA	p.Glu1975fs	N/A
P0007	*ALMS1*[*]	chr2:73448312TG>T	c.1786delG	p.Glu596fs	N/A
P0007	*ALMS1*[*]	chr2:73572849C>T	c.10972C>T	p.Arg3658*	N/A
A0018[†]	*BAG3*	chr10:119670037C>T	c.367C>T	p.Arg123*	P/LP
A0168[†]	*BAG3*	chr10:119670037C>T	c.367C>T	p.Arg123*	P/LP
A0043	*DSP*	chr6:7542086G>C	c.170+1G>C	N/A	N/A
A0048	*DSP*	chr6:7580707GA>G	c.4518delA	p.Arg1506fs	LP
A0083	*DSP*	chr6:7579385C>G	c.3195C>G	p.Tyr1065*	P
P0038	*FLNC*	chr7:128830943G>GT	c.306_307insT	p.Ala103fs	N/A
A0096	*LMNA*	chr1:156134949G>T	c.784G>T	p.Glu262*	LP
A0131	*LMNA*	chr1:156115015G>T	c.97G>T	p.Glu33*	N/A
A0169	*LMNA*	chr1:156136956GA>G	c.1418delA	p.Asn473fs	N/A
A0127	*MYBPC3*	chr11:47351507T>C	c.26-2A>G	N/A	P/LP
A0181	*RBM20*	chr10:110799919G>A	c.1800+1G>A	N/A	VUS
A0112	*SCN5A*	chr3:38575391C>T	c.3572G>A	p.Trp1191*	N/A
P0014[‡]	*TNNI3*	chr19:55157095G>GC	c.62dupG	p.Arg22fs	N/A
A0023	*TTN*	chr2:178738202A>AAG	c.13160_13161dupCT	p.Ser4388fs	N/A
A0027	*TTN*	chr2:178557649G>C	c.60510C>G	p.Tyr20170*	LP
A0033	*TTN*	chr2:178630272AT>A	c.17054delA	p.Asn5685fs	N/A
A0044	*TTN*	chr2:178579775CT>C	c.40226delA	p.Lys13409fs	N/A
A0052	*TTN*	chr2:178552121T>TA	c.63583dupT	p.Tyr21195fs	LP
A0082	*TTN*	chr2:178574530G>A	c.44407C>T	p.Arg14803*	P/LP
A0087	*TTN*	chr2:178584831G>A	c.37615C>T	p.Arg12539*	N/A
A0092	*TTN*	chr2:178619888C>T	c.19235-1G>A	N/A	N/A
A0102	*TTN*	chr2:178574740G>A	c.44197C>T	p.Gln14733*	N/A
A0103	*TTN*	chr2:178591466CT>C	c.33063delA	p.Gly11022fs	N/A
A0132	*TTN*	chr2:178564096G>A	c.54841C>T	p.Gln18281*	P/LP
A0134	*TTN*	chr2:178561756G>A	c.57181C>T	p.Gln19061*	LP
A0138	*TTN*	chr2:178614754C>G	c.21566-1G>C	N/A	LP
A0161	*TTN*	chr2:178567551GA>G	c.51385delT	p.Ser17129fs	N/A
A0188	*TTN*	chr2:178592916CAG>C	c.32006_32007delCT	p.Pro10669fs	LP
A0191	*TTN*	chr2:178559619A>C	c.59318T>G	p.Leu19773*	N/A
A0193	*TTN*	chr2:178552121T>TA	c.63583dupT	p.Tyr21195fs	LP

[*]Autosomal recessive inheritance, [†]A0018 and A0168 are related individuals, [‡]Also has a *TNNT3* damaging-missense variant.
AA: amino acid, CDS: coding sequence, LP: likely pathogenic, N/A: not available, P: pathogenic

Supplemental Table IV. Damaging-missense variants in definite cardiomyopathy-associated genes in 99 DCM samples

ID	Gene	GRCh38 coordinate	CDS change	AA change	ClinVar
A0020	*LMNA*	chr1:156115072C>G	c.154C>G	p.Leu52Val	LP
P0011*	*LMNA*	chr1:156135244G>A	c.868G>A	p.Glu290Lys	C
P0024*	*LMNA*	chr1:156135244G>A	c.868G>A	p.Glu290Lys	C
A0056	*MYH7*	chr14:23429266C>A	c.1220G>T	p.Gly407Val	LP
P0014†	*TNNT2*	chr1:201364365C>T	c.392G>A	p.Arg131Gln	P
P0037	*TNNT2*	chr1:201364336G>A	c.421C>T	p.Arg141Trp	P
P0042	*TPM1*	chr15:63062263G>A	c.688G>A	p.Asp230Asn	P

*P0011 and P0024 are unrelated individuals, †Also has a *TNNI3* loss-of-function variant.
AA: amino acid, C: conflicting interpretations of pathogenicity, CDS: coding sequence, LP: likely pathogenic, P: pathogenic

Supplemental Table V. Structural variants in definite cardiomyopathy-associated genes in 99 DCM samples

ID	Gene	Chr	Start	End	Length	Variant impact
A0036	*TTN*	2	178607725	178611540	3815	Out-of-frame deletion based on WGS analysis
A0097	*BAG3*	10	119649294	119653539	4245	Deletion of promoter and exon 1 based on WGS analysis and haploinsufficiency based on RNA-seq analysis
A0187	*DMD*	X	31625488	32117000	491512	In-frame deletion and addition of GAG (Glu) based on RNA-seq analysis

WGS: whole genome sequencing

Supplemental Table VI. Splicing variants in definite cardiomyopathy-associated genes in 99 DCM samples

ID	Gene	GRCh38 coordinate	CDS change	Predicted effect	ClinVar	Variant impact
A0035	*LAMP2*	chrX:120442595T>C	c.928+4A>G	Donor loss	VUS	Out-of-frame deletion based on RNA-seq analysis
A0014	*LMNA*	chr1:156136110C>T	c.1146C>T	Donor gain	P/LP	Out-of-frame deletion based on RNA-seq analysis

CDS: coding sequence, LP: likely pathogenic, P: pathogenic, VUS: variant of unknown significance

Supplemental Table VII. Rare loss-of-function, damaging-missense, and splicing variants in DCM-associated genes with moderate and limited evidence found in explant DCM tissues

ID	Gene	GRCh38 coordinate	CDS change	AA change	ClinVar
A0172	*EYA4*	chr6:133515399C>T	c.1580C>T	p.Thr527Ile	N/A
A0110	*MYH6*	chr14:23396316C>T	c.2397G>A	p.Met799Ile	VUS
P0011	*MYH6*	chr14:23393752C>T	c.2842G>A	p.Glu948Lys	VUS
A0045	*NEBL*	chr10:20850508T>C	c.1009-6A>G	N/A	N/A
A0205	*OBSCN*	chr1:228279410C>T	c.7362C>T	p.Gly2454Gly	N/A
A0119	*SGCD*	chr5:156589234A>C	c.298A>C	p.Asn100His	N/A
A0147	*TBX20*	chr7:35249976C>T	c.355G>A	p.Glu119Lys	N/A

AA: amino acid, CDS: coding sequence, LP: likely pathogenic, N/A: not available, P: pathogenic

Supplemental Table VIII. Pathogenic variants in definite cardiomyopathy-associated genes in 18 HCM samples

ID	Gene	GRCh38 coordinate	CDS change	AA change	ClinVar	Variant type
A0076	*MYBPC3*	chr11:47337729A>AC	c.2373dupG	p.Trp792fs	P	Loss-of-function
A0117	*MYBPC3*	chr11:47332116TTGG>T	c.772G>A	p.Glu258Lys	P/LP	Damaging-missense
A0126	*MYBPC3*	chr11:47348449G>T	c.2149-1 G>C	N/A	P	Loss-of-function
A0142	*MYBPC3*	chr11:47337729A>AC	c.3767_3769 delCCA	p.Thr1256del	LP	Damaging-missense
A0207	*MYBPC3*	chr11:47348424C>T	c.747C>A	p.Cys249*	P/LP	Loss-of-function
A0068	*MYH7*	chr14:23429005G>A	c.1357C>T	p.Arg453Cys	P (HCM)	Damaging-missense
A0070	*MYH7*	chr14:23425980C>G	c.2146G>C	p.Gly716Arg	P (HCM)	Damaging-missense
P0019	*RIT1*	chr1:155904798G>C	c.170C>G	p.Ala57Gly	P	Damaging-missense
A0108	*TNNT2*	chr1:201365297C>T	c.305G>A	p.Arg102Gln	P/LP (HCM)	Damaging-missense

AA: amino acid, CDS: coding sequence, LP: likely pathogenic, N/A: not available, P: pathogenic

Supplemental Table IX. Pathogenic variants in definite cardiomyopathy-associated genes in 3 ACM samples

ID	Gene	GRCh38 coordinate	CDS change	AA change	ClinVar	Variant type
A0141	*PKP2*	chr12:32877917GA>G	c.962delT	p.Val321fs	N/A	Loss-of-function
A0099	*TMEM43*	chr3:14141665C>T	c.1073C>T	p.Ser358Leu	P	Damaging-missense

AA: amino acid, CDS: coding sequence, N/A: not available, P: pathogenic

Supplemental Table X. Pathogenic variants in definite cardiomyopathy-associated genes in 2 RCM samples

ID	Gene	GRCh38 coordinate	CDS change	AA change	ClinVar	Variant type
P0044	*MYH7*	chr14:23424935G>A	c.2513C>T	p.Pro838Leu	P	Damaging-missense

AA: amino acid, CDS: coding sequence, P: pathogenic

Supplemental Table XI. The number of pathogenic variants in 48 genes tested in Verdonschot et al.[34]

Gene	End-stage DCM cases			DCM cases at all stages		
	PVpos	Total	Prevalence (%)	PVpos	Total	Prevalence (%)
ACTC1	0	85	0.00	2	689	0.29
ACTN2	0	85	0.00	0	689	0.00
ANKRD1	0	85	0.00	0	689	0.00
BAG3	3	85	3.53	1	689	0.15
CALR3	0	85	0.00	0	689	0.00
CAV3	0	85	0.00	0	689	0.00
CRYAB	0	85	0.00	0	689	0.00
CSRP3	0	85	0.00	0	689	0.00
CTNNA3	1	85	1.18	0	689	0.00
DES	0	85	0.00	0	689	0.00
DSC2	0	85	0.00	0	689	0.00
DSG2	0	85	0.00	0	689	0.00
DSP	3	85	3.53	4	689	0.58
EMD	0	85	0.00	1	689	0.15
FHL1	0	85	0.00	0	689	0.00
FLNC	1	85	1.18	3	172	1.74
GLA	0	85	0.00	0	689	0.00
JPH2	0	85	0.00	0	689	0.00
JUP	0	85	0.00	0	689	0.00
LAMA4	0	85	0.00	0	689	0.00
LAMP2	1	85	1.18	0	689	0.00
LDB3	0	85	0.00	0	689	0.00
LMNA	5	85	5.88	21*	689	3.05
MIB1	0	85	0.00	0	689	0.00
MYBPC3	1	85	1.18	0	689	0.00
MYH6	0	85	0.00	0	689	0.00
MYH7	1	85	1.18	7	689	1.02
MYL2	0	85	0.00	1[†]	689	0.15
MYL3	0	85	0.00	0	689	0.00
MYOZ2	0	85	0.00	0	689	0.00
MYPN	0	85	0.00	0	689	0.00
NEXN	0	85	0.00	2	689	0.29
PKP2	0	85	0.00	0	689	0.00
PLN	0	85	0.00	3	689	0.44

PRDM16	0	85	0.00	0	689	0.00
PRKAG2	0	85	0.00	0	689	0.00
RBM20	1	85	1.18	5	689	0.73
SCN5A	1	85	1.18	1	689	0.15
TAZ	0	85	0.00	0	689	0.00
TCAP	0	85	0.00	0	689	0.00
TMEM43	0	85	0.00	0	689	0.00
TNNC1	0	85	0.00	1	689	0.15
TNNI3	0	85	0.00	0	689	0.00
TNNT2	0	85	0.00	4	689	0.58
TPM1	0	85	0.00	5	689	0.73
TTN	18	85	21.18	66‡	689	9.58
TTR	0	85	0.00	2	689	0.29
VCL	0	85	0.00	0	689	0.00
Total	36	85	42.35	129	689	18.72

*one patient also with likely pathogenic TTN variant, †homozygous, ‡one patient also with likely pathogenic TNNT2 variant.

Supplemental Table XII. The number of pathogenic variants in 35 genes tested in Morales et al.[35]

Gene	End-stage DCM cases			DCM cases at all stages		
	PVpos	Total	Prevalence (%)	PVpos	Total	Prevalence (%)
ABCC9	0	85	0.00	0	97	0.00
ACTC1	0	85	0.00	0	97	0.00
ACTN2	0	85	0.00	0	97	0.00
ANKRD1	0	85	0.00	0	97	0.00
BAG3	3	85	3.53	1	97	1.03
CRYAB	0	85	0.00	0	97	0.00
CSRP3	0	85	0.00	0	97	0.00
DES	0	85	0.00	0	97	0.00
DSG2	0	85	0.00	0	97	0.00
DSP	3	85	3.53	0	97	0.00
EYA4	0	85	0.00	0	97	0.00
FLNC	0	85	0.00	2	97	2.06
ILK	0	85	0.00	0	97	0.00
LAMA4	0	85	0.00	0	97	0.00
LDB3	0	85	0.00	0	97	0.00
LMNA	5	85	5.88	2	97	2.06
MYBPC3	1	85	1.18	0	97	0.00

MYH6	0	85	0.00	0	97	0.00
MYH7	1	85	1.18	0	97	0.00
MYPN	0	85	0.00	0	97	0.00
NEBL	1	85	1.18	0	97	0.00
NEXN	0	85	0.00	0	97	0.00
PDLIM3	0	85	0.00	0	97	0.00
PKP2	0	85	0.00	0	97	0.00
PLN	0	85	0.00	0	97	0.00
RBM20	1	85	1.18	1	97	1.03
SCN5A	1	85	1.18	0	97	0.00
SGCD	0	85	0.00	0	97	0.00
TCAP	0	85	0.00	0	97	0.00
TNNC1	0	85	0.00	0	97	0.00
TNNI3	0	85	0.00	0	97	0.00
TNNT2	0	85	0.00	1	97	1.03
TPM1	0	85	0.00	0	97	0.00
TTN	18	85	21.18	8	97	8.25
VCL	0	85	0.00	0	97	0.00
Total	34	85	40.00	15	97	15.46

Supplemental Table XIII. The number of pathogenic variants in 57 genes tested in Mazzarotto et al.[6]

Gene	End-stage DCM cases			DCM cases at all stages		
	PVpos	Total	Prevalence (%)	PVpos	Total	Prevalence (%)
ABCC9	0	85	0.00	2	1040	0.19
ACTA1	0	85	0.00	0	1040	0.00
ACTC1	0	85	0.00	0	1040	0.00
ACTN2	0	85	0.00	0	1040	0.00
ANKRD1	0	85	0.00	1	1040	0.10
BAG3	3	85	3.53	3	1040	0.29
CASQ2	0	85	0.00	1	1040	0.10
CRYAB	0	85	0.00	0	1040	0.00
CSRP3	0	85	0.00	0	1040	0.00
DES	0	85	0.00	0	1040	0.00
DMD	1	85	1.18	0	1040	0.00
DNAJC19	0	85	0.00	0	1040	0.00
DOLK	0	85	0.00	2	1040	0.19
DSC2	0	85	0.00	0	1040	0.00

DSG2	0	85	0.00	3	1040	0.29
DSP	3	85	3.53	15	1040	1.44
EMD	0	85	0.00	0	1040	0.00
FKTN	0	85	0.00	1	1040	0.10
GATAD1	0	85	0.00	0	1040	0.00
ILK	0	85	0.00	0	1040	0.00
JUP	0	85	0.00	0	1040	0.00
LAMA2	0	85	0.00	1	1040	0.10
LAMA4	0	85	0.00	1	1040	0.10
LAMP2	1	85	1.18	1	1040	0.10
LDB3	0	85	0.00	1	1040	0.10
LMNA	5	85	5.88	8	1040	0.77
MURC	0	85	0.00	0	1040	0.00
MYBPC3	1	85	1.18	3	1040	0.29
MYH6	0	85	0.00	0	1040	0.00
MYH7	1	85	1.18	3	1040	0.29
MYL2	0	85	0.00	0	1040	0.00
MYPN	0	85	0.00	1	1040	0.10
NEXN	0	85	0.00	0	1040	0.00
NKX2-5	0	85	0.00	0	1040	0.00
NPPA	0	85	0.00	0	1040	0.00
PDLIM3	0	85	0.00	0	1040	0.00
PKP2	0	85	0.00	2	1040	0.19
PLN	0	85	0.00	0	1040	0.00
PRDM16	0	85	0.00	1	1040	0.10
RAF1	0	85	0.00	0	1040	0.00
RBM20	1	85	1.18	0	1040	0.00
SCN5A	1	85	1.18	4	1040	0.38
SGCB	0	85	0.00	0	1040	0.00
SGCD	0	85	0.00	0	1040	0.00
TAZ	0	85	0.00	0	1040	0.00
TBX20	0	85	0.00	0	1040	0.00
TBX5	1	85	1.18	1	1040	0.10
TCAP	0	85	0.00	0	1040	0.00
TMPO	0	85	0.00	0	1040	0.00
TNNC1	0	85	0.00	0	1040	0.00
TNNI3	0	85	0.00	1	1040	0.10
TNNT2	0	85	0.00	5	1040	0.48
TPM1	0	85	0.00	1	1040	0.10

TTN	18	85	21.18	118	1040	11.35
TXNRD2	0	85	0.00	2	1040	0.19
VCL	0	85	0.00	1	1040	0.10
ZBTB17	0	85	0.00	0	1040	0.00
Total	36	85	42.35	183	1040	17.60
